# Publisher Correction: Lineage-specific, fast-evolving GATA-like gene regulates zygotic gene activation to promote endoderm specification and pattern formation in the Theridiidae spider

**DOI:** 10.1186/s12915-022-01494-x

**Published:** 2022-12-17

**Authors:** Sawa Iwasaki-Yokozawa, Ryota Nanjo, Yasuko Akiyama-Oda, Hiroki Oda

**Affiliations:** 1 Laboratory of Evolutionary Cell and Developmental Biology, JT Biohistory Research Hall, Takatsuki, Osaka 569-1125 Japan; 2 Department of Biological Sciences, Graduate School of Science, Osaka University, Toyonaka, Japan; 3 PRESTO, Japan Science and Technology Agency, Kawaguchi, Saitama 332-0012 Japan; 4 Department of Microbiology and Infection Control, Faculty of Medicine, Osaka Medical and Pharmaceutical University, Takatsuki, Japan

**Publisher Correction: BMC Biol 20, 223 (2022)**

**https://doi.org/10.1186/s12915-022-01421-0**

The original article [1] erroneously presented the supplementary materials in accordance with their respective citations; the supplementary files are now correctly presented and cited.
